# Solid Swallow Examination During High Resolution Manometry and EGJ-Distensibility Help Identify Esophageal Outflow Obstruction in Non-obstructive Dysphagia

**DOI:** 10.1007/s00455-021-10260-0

**Published:** 2021-02-20

**Authors:** Fritz Ruprecht Murray, Lara Maria Fischbach, Valeria Schindler, Larissa Schnurre, Juliane Marie Hente, Aurora Tatu, Daniel Pohl

**Affiliations:** Division of Gastroenterology and Hepatology, University Hospital Zurich, Raemistrasse 100, 8091 Zurich, Switzerland

**Keywords:** Solid swallow high-resolution manometry, FLIP, Esophagogastric outlet obstruction, Non-obstructive dysphagia

## Abstract

Single water swallow (SWS) high-resolution manometry (HRM) may miss relevant esophageal motility disorders. Solid test meal (STM) during HRM and lately the functional lumen imaging probe (FLIP) have been shown to be of diagnostic value in the assessment of motility disorders. We aimed to assess the diagnostic yield of STM and FLIP in non-obstructive dysphagia (NOD). Patients assessed for dysphagia with both HRM and FLIP between April 2016 and August 2019 were analyzed for signs of non-obstructive EGJ outflow obstruction (EGJOO) according to Chicago Classification 3.0 (CCv3) and CC adapted for the use with solid swallows (CC-S), followed by an individual group-specific analysis. Five subjects without dysphagia served as control group. Standard HRM- and FLIP-values as well as esophagograms and Eckardt Scores were analyzed. Forty-two patients were identified (male/female, 14/36, median age 62). Twenty-five (59.5%) were diagnosed with EGJOO during STM only (= SWS-negative patients; CC-S). The EGJ distensibility index (EGJ-DI) of symptomatic patients was significantly lower compared to the control group (*p* = 0.006). EGJ-DI was < 3mm^2^/mmHg in 67% and 88% of patients diagnosed according to CC-S and CCv3, respectively. The IRP during STM showed a significant association to the corresponding EGJ-DI values (*p* < 0.001). Seventy-six percent of patients received treatment because of additional STM evaluation with a favorable clinical response rate of 89%. STM and FLIP identify EGJOO in symptomatic patients with normal SWS during HRM. STM resembles an inexpensive and clinically meaningful option to diagnose motility disorders and helps to select patients for interventional treatment.

## Introduction

Achalasia and esophagogastric junction outlet obstruction (EGJOO) are major motility disorders with impaired relaxation of the lower esophageal sphincter (LES). Achalasia is a rare but well-characterized disease, caused by a degeneration or dysfunction of inhibitory postganglionic neurons in the distal esophagus [1]. In contrast, EGJOO is less clearly characterized and may be caused by anatomic obstacles, such as a large hiatal hernia, distal esophageal stricture or fundoplication; can be of functional origin or may represent an early stage of achalasia [2]. Furthermore, EGJOO has been shown to be associated with opioid and excessive alcohol consumption [3].

To date, the metrics of single water swallows (SWS) collected during high-resolution manometry (HRM) are routinely used for the diagnosis of these disorders, based on the Chicago Classification Version 3.0 (CCv3) [4]. However, HRM may miss motility disorders in patients with non-obstructive dysphagia (NOD) [5, 6], leaving them without a definite diagnosis and thus uncertain further management. The inclusion of a standardized solid test meal (STM) to HRM has been shown to increase the diagnostic sensitivity of HRM with the possibility of establishing motility disorders with the observations made during STM [7]. In response to this, an adaptation of the Chicago Classification (CC) adapted for the use with solid swallows (CC-S) was recently suggested, proposing a diagnostic hierarchy for major motility disorders during STM [8]. In this classification an integrated relaxation pressure (IRP) > 25 mmHg in ≥ 2 swallows was defined as pathologic.

The endoluminal functional lumen imaging probe (FLIP) is being increasingly used as an additional diagnostic tool for the assessment of esophageal function, including motility [9]. In addition, FLIP provides comprehensive information regarding LES physiology by measuring the EGJ distensibility index (EGJ-DI), a function of the EGJ cross-sectional area (CSA) and the FLIP intra-balloon pressure [10–14]. Recently Triggs et al. published a study showing FLIP to be useful to identify EGJOO patients who benefit most likely from achalasia-type treatment [14]. While focusing on treatment outcomes, all included patients were diagnosed according to single water swallows only, leaving the question of how to proceed with the relevant proportion of dysphagia patients without findings during single swallow HRM unanswered.

In recent years, FLIP studies have been able to demonstrate that a pathologic EGJ-DI identifies achalasia in patients with suggestive radiological and clinical features not fulfilling formal HRM criteria for diagnosis of achalasia [15]. Until now, EGJOO has been largely neglected in EGJ-DI studies, especially regarding a potential diagnostic benefit in symptomatic patients not fulfilling CCv3 criteria in HRM.

The aim of this study was to further assess the diagnostic yield of both STM and FLIP findings in non-achalasia patients with symptomatic NOD. To the best of our knowledge this is the first study, presenting clinically relevant outcomes of additional diagnostic procedures resulting in achalasia-type treatment in CCv3 non-obstructive but symptomatic patients. In addition, the results of this study for the first time identify EGJ-DI values in EGJOO patients.

## Materials and Methods

### Patients

Ninety-three patients with clinically relevant dysphagia, diagnosed with EGJOO either during SWS according to CCv3 or during STM between April 2016 and August 2019 were analyzed. For each patient, age, sex, current medication, results of previous upper endoscopy, medical history, clinical symptoms and Eckardt Score (ES) were documented prior to HRM. All patients were assessed with both HRM, routinely including SWS and STM, and FLIP according to the routine clinical management of our clinic. Further inclusion criteria were age ≥ 18 years and signed general informed consent. Patients diagnosed with achalasia according to CCv3 were not considered. Fifty-one patients were excluded for the following reasons: obstructive dysphagia (Schatzki, *n* = 1; eosinophilic eosophagitis, *n* = 1; large axial hernia, *n* = 1; previous mediastinitis with fibrosis, *n* = 1; distal eosophageal spasm, *n* = 2), previous interventions at the EGJ (fundoplication, *n* = 13; previous dilation, *n* = 11), upper GI surgery (*n* = 1), current opiate intake, *n* = 13) and/or pulmonary (*n* = 1) and gastric (*n* = 1) cancer; 5 other patients were excluded for rejected general informed consent. In addition to symptomatic patients, datasets of five patients without symptomatic dysphagia with an ES of 0 points who had received both HRM and FLIP for other reasons (e.g. pyloric dysfunction as a clinical lead for gastroparesis, gastric belching or gastroesophageal reflux disease) were documented. During these investigations, the EGJ was routinely examined and thus clinical data of non-dysphagia patients was gathered. All investigations were conducted according to the standard clinical workup of symptomatic dysphagia of our institution. Datasets were analyzed after approval of the Zurich Ethics Committee (KEK-Nr: 2017-00930).

Prior to further analysis, patients were separated into two groups: the first group included patients with pathologic patterns during STM according to CC-S (STM-group). All of these patients had IRP values of < 15 mmHg during SWS (= SWS-negative) and at least 2 swallows with IRP > 25 mmHg during STM and a normal swallow pattern during SWS. In contrast, group two (SWS-group), meant to function as a comparative group, included patients diagnosed with EGJOO during SWS according to CCv3 (IRP ≥ 15 mmHg and non-achalasia swallow pattern). All patients considered pathologic during SWS also met criteria regarded as pathologic during STM. An additional third group, meant to function as a control group, consisted of patients without symptomatic dysphagia (ES = 0). All calculated IRP values both in SWS and STM were < 15 mmHg. No obstructive, spastic or hypercontractile swallow patterns were demarcated during SWS or STM. Of note, patients in the control group were considerably younger than those included into the STM- and SWS-group.

### Procedures

The analysis of all HRM and STM data was conducted by members of the Functional Diagnostic Department of the University Hospital Zurich (all authors) with a final review of the head of the department (DP). All endoscopic procedures (FLIP) were conducted by one endoscopist (DP).

### Standard HRM Procedure

HRM was performed with a 36 channel solid-state HRM catheter (Manoscan 360, Sierra Scientific Instruments, Given Imaging, Los Angeles, CA, USA). The application, calibration procedure and the post study-preparation procedure of the data has been described in detail elsewhere [16, 17]. The applied protocol included ten SWS with a volume of 10 ml administered in semi-upright position, followed by a rapid drink challenge of 100 ml. Afterwards, a STM was consumed by each patient, followed by another rapid drink challenge. All HRM studies were analyzed with ManoView ESO version 3.0.1., followed by classification according to CCv3 [4].

### Procedure and Analysis of STM

STM consisted of 200 g of freshly cooked salted plain rice (Uncle Bens, Brussels, Belgium; 270 kcal). STM analysis was conducted in accordance to previous studies and results were classified according to CC-S [7, 8, 18]. For each patient, individual frames were manually inserted into the recorded STM period (ManoView ESO version 3.0.1) to calculate the IRP for every esophageal contraction during STM. According to previously published literature, IRP values of 25 mmHg in ≥ 2 solid swallows during STM were considered pathologic, leading to the diagnosis of EGJOO [8].

### FLIP Investigation EGJ

EGJ-DI measurements were performed with FLIP probe system (EndoFLIP®, Crospon Ltd, Galway, Ireland) during upper endoscopy. The characteristics, technical details and exact application of this device are described elsewhere [13, 15, 19, 20]. In short, the FLIP was introduced during upper endoscopy in close proximity of the EGJ. After retracting the endoscope, the final positioning of the balloon was guided by the FLIP technology showing a narrowing of the balloon. Two different device sizes were used due to logistic reasons: EndoFLIP-325 (EF-325) and EndoFLIP-322 (EF-322). The characteristics, technical details and exact procedure of this device are described elsewhere [13, 15, 19]. EF-325 investigations were started at a catheter volume of 30 ml and the EF-322 at 40 ml. Volumes were increased in 10 ml steps to a maximum of 50 ml (EF-325) and 70 ml (EF-322).. During examination, cross sectional area (CSA), diameter, EGJ-DI and balloon pressure were recorded following a standardized protocol. Depending on the catheter type, different filling volumes for the reference values were used in order to guarantee an adequate balloon pressure (> 20 mmHg) in accordance to published normative values [13, 19, 21]: 40 ml and 60 ml for the EF-325 and -322, respectively. An EGJ-DI of < 3mm^2^/mmHg with a balloon pressure of at least 20 mmHg was considered pathologic [13, 21].

### Timed Barium Esophagogram and Eckardt Score

Results of pre-and post-interventional timed barium esophagograms (TBE) were added to the analysis. TBE images were taken in upright position 1, 3 and 5 min after ingestion of a standard low-density liquid barium swallow. In case of stasis, the height of the barium column was measured at each respective time point. A reduction of the column height was adopted as positive therapy response. For clinical follow-up, ES was reported pre- and post-interventionally. An improvement in the ES was considered positive as either a reduction of 3 points to baseline or a total ES below 3 points.

### General Information Regarding Treatment Decisions

Up to the analysis of the underlying study, treatment decisions in our clinic in case of EGJOO depended on an analysis of varies diagnostic parameters consisting of ES, HRM (SWS + STM), TBE and the individual desire of patients. Due to the lack of normative values and clinical evidence, FLIP metrics were not part of treatment decisions of patients included in this manuscript.

### Endpoints

The primary endpoint was the assessment of the diagnostic yield of both STM and FLIP findings in non-achalasia patients with symptomatic NOD as follows: Corresponding STM-IRP and EGJ-DI values were analyzed, with focus on SWS-negative patients with pathologic STM findings according to CC-S. In addition, EGJ-DI values of symptomatic patients and an asymptomatic control group were compared.

The secondary endpoint was an analysis of FLIP and STM findings in SWS-negative patients in regard to therapeutic consequences, focusing on TBE and ES.

### Statistical Analysis

Data analysis was performed with R version 3.5.2 (R Foundation for Statistical Computing, Vienna, Austria, 2018-12-20). To compare data of different groups Wilcoxon signed-rank test as well as generalized linear models were used. In order to analyze the relation between STM-IRP and distensibility, linear regression analysis was performed. A *p *value < 0.05 was regarded as significant. The continuous data is displayed in median (range) or number (percent of total).

## Results

### Patient and Group-Specific Characteristics

All included patients were primarily assessed with HRM for dysphagia, followed by an additional examination with FLIP. A total of 42 patients (men 22/42 [47%], median age, 62 years [range 24–88]) matched the above mentioned inclusion criteria. All group specific characteristics are displayed in Table 1.

**Table 1:** Group specific characteristics

	STM-group	SWS-group	Control-group
Characteristics			
Number of patients	25	17	5
Gender (m/w)	11/25	7/17	4/5
Age (median, range)	62, 28–88	72, 47–82	28, 24–53
IRP SWS (mmHg)			
Median	9.0	19.7	4.4
Range	4.2–14.8	15.8–38.2	0.1–7.8
IRP STM (mmHg)			
Median	21.9	26.7	2.5
Range	15.1–35.1	17.8–35.3	1–8.9
Type FLIP used			
EF-325	18	17	4
EF-322	7	0	1
EGJ-DI (mm^2^/mmHg)			
EF-325 at 40 ml (median, range)	2.1, 0.3–7.0	1.8, 0.7–5.6	6.9, 3.6–9.4
EF-322 at 60 ml (median, range)	1.5, 0.8–2.7		3.1
EGJ-DI < 3mm^2^/mmHg	19 (67%)	15 (88%)	0
Eckardt Score			
Median	3	4	0
Range	1–10	2–9	
TBE (number of patients)^a^			
Abnormal motility	4	5	
Stasis	14	9	
After 3 min (median hight in cm, range)	14 (5, 2–11)	9 (3, 1–6.5)	
After 5 min (median hight in cm, range)	7 (5, 0.5–11)	1 (3)	

*IRP* integrated relaxation pressure, *SWS *single water swallows, *STM *standard test meal, *FLIP *functional lumen imaging probe, *EF *EndoFLIP, *EGJ-DI* EGJ distensibility index, *TBE *timed barium esophagogram

^a^Only 23/25 patients in STM-group and no patient in the control-group received TBE

### Yield of Additional Analysis of STM in Patients with Normal HRM According to CCv3

All (100%) patients included in the STM-group (*n* = 25) were classified as normal during SWS according to CCv3 (= SWS-negative). Accordingly, none of these patients (all of them symptomatic) would have been considered for achalasia type treatment if only accessed with conventional SWS HRM.

### Association of STM-IRP and EGJ-DI

Linear regression analysis of the IRP during STM in all investigated patients (STM-group + SWS-group + control group) showed a statistically significant association to the corresponding EGJ-DI values (*p* < 0.001). Statistically, a decrease of 1.0mm^2^/mmHg in EGJ-DI was associated with an increase of 2.2 mmHg in STM-IRP values.

### Comparison of EGJ-DI Between Groups

**Fig. 1:**
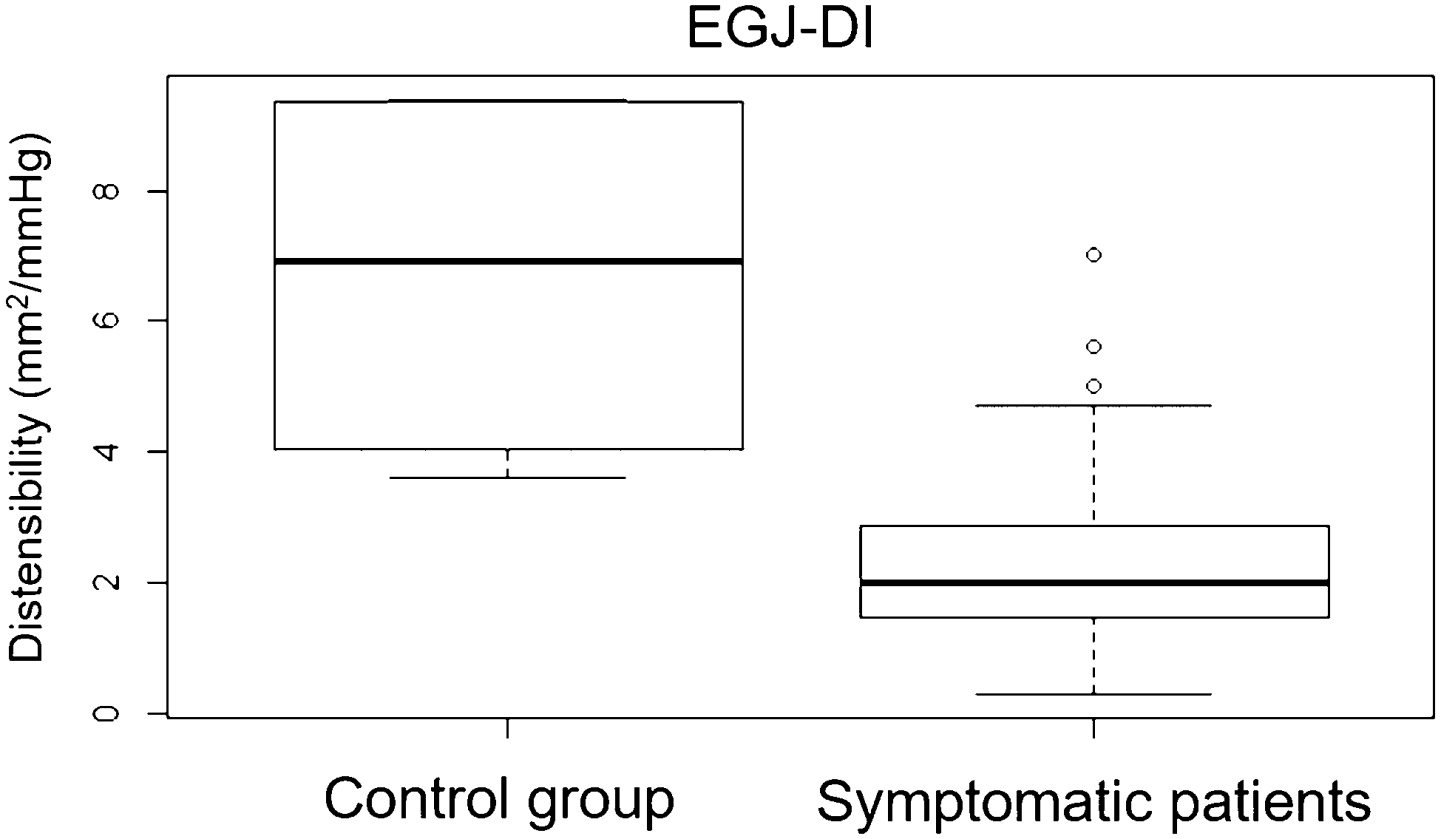
EGJ-DI results in control group

**Fig. 2:**
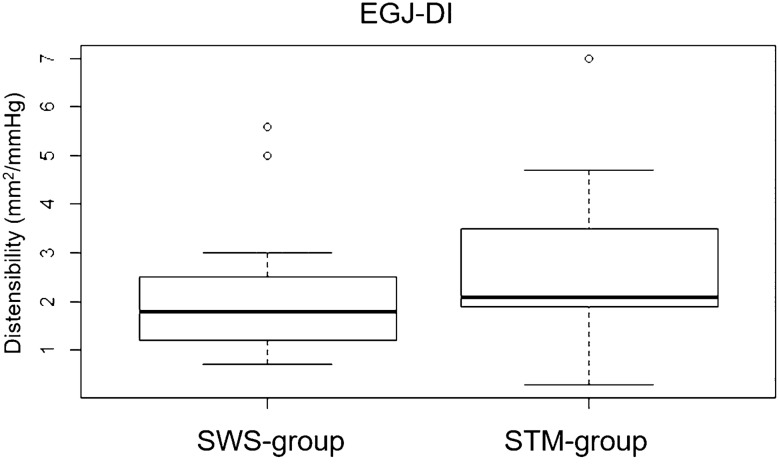
EGJ-DI results in symptomatic patiens (SWS-group and STM-group)

**Fig. 3:**
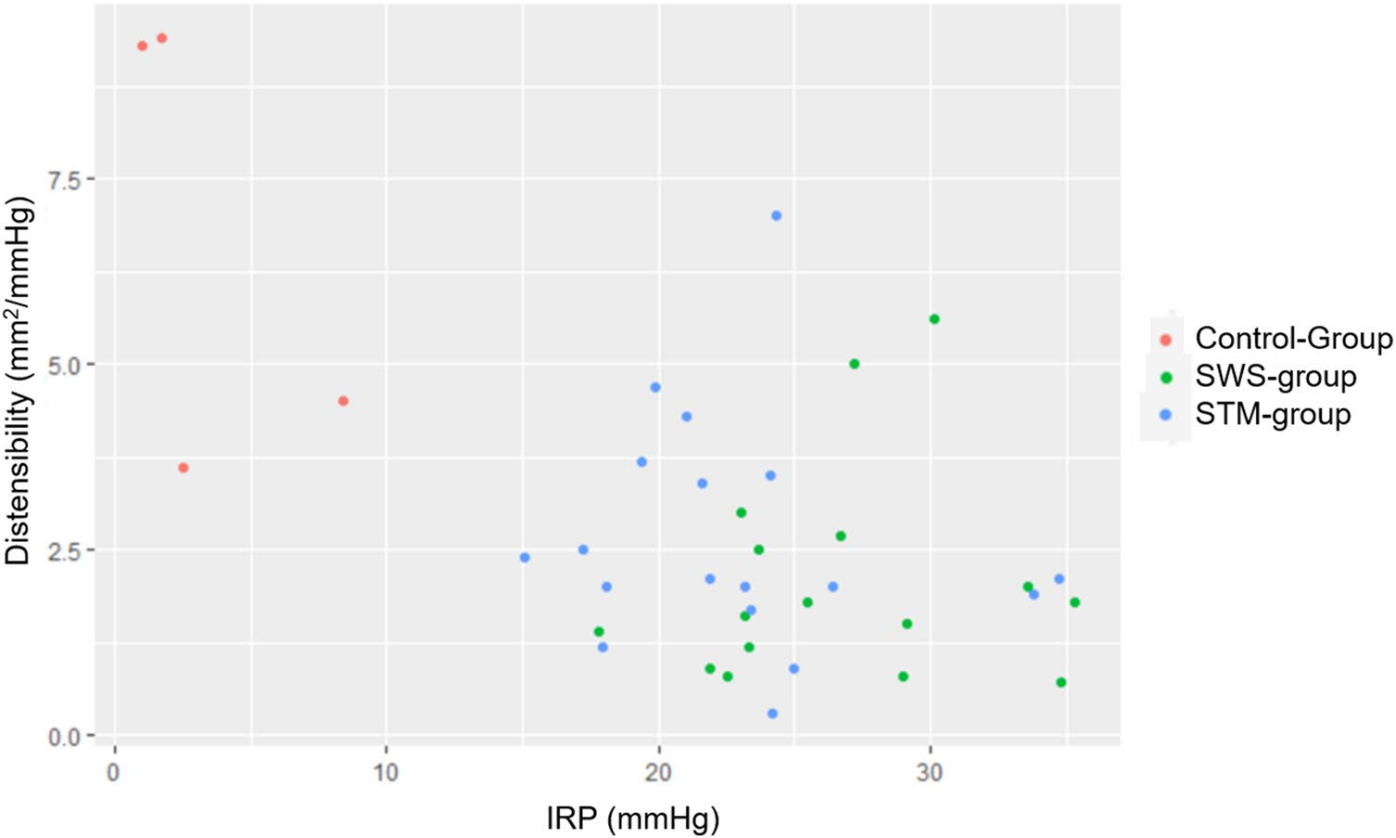
Association of IRP values and EGJ-DI

EGJ-DI in symptomatic patients (STM- and SWS-group combined) was significantly lower (median, 2.0mm^2^/mmHg; range 0.3–7.0mm^2^/mmHg) than in the control group (median 6.9mm^2^/mmHg; range 3.6–9.4mm^2^/mmHg) (*p* = 0.006, shown in Fig. 1). Values of the STM- (median 2.1 mm^2^/mmHg; range 0.3–7.0 mm^2^/mmHg) and SWS-group (median, 1.8 mm^2^/mmHg; range 0.7–5.6 mm^2^/mmHg), however, did not significantly differ from one another (*p* = 0.109, shown in Fig. 2). Figure 3 illustrates the association between IRP values and EGJ-DI in the three subgroups.

### Achalasia-Type Treatment in SWS-Group

In the SWS-group 9/17 patients (53%) received interventional treatment with the EsoFLIP device, which has been implemented in the management of the treatment of EGJ disorders in our clinic (first results of the treatment of achalasia patients have been published; [22]). The median distensibility of these patients was 1.6 mm2/mmHg (range 0.7–2.0mm^2^/mmHg). Pre-interventional TBE showed stasis in 6/9 patients (67%) and 3/9 (33%) showed abnormal motility. All patients were dilated once, until the median diameter of 26 mm (range 19–26 mm). Of these 9 patients, 6 received a second EsoFLIP-controlled dilatation (median diameter, 27 mm; range 26–29 mm). Clinical and radiological follow-up was performed in a median of 10 days (range 4–33) after treatment. Post-interventionally, the median ES improved from 4 (range 3–9) to 0 (range 0–2) in all treated patients. TBE, performed in 8/9 after the first and in 4/6 patients after the second dilation, showed a reduction of stasis or normal motility patterns in all treated patients. Eight patients did not receive achalasia-type treatment. Three (37.5%) had a normal distensibility (≥ 3mm^2^/mmHg), 3/8 (37.5%) had a reduced distensibility but normal TBE and clinically improved after a proton pump inhibitor trial, and 2/8 (25%) declined treatment (despite reduced distensibility and abnormal TBE).

### Consequences of Motility Disorder Demarcation During STM

Nineteen patients in the STM-group (76%) received achalasia-type treatment as a consequence of pathologic findings during STM. The majority (16/19, 84%) received at least one balloon dilation. Most (15/16, 94%) of these dilations were conducted with the EsoFLIP device. Of these, 7 (7/15) received a second EsoFLIP-controlled dilation and three (3/15) were additionally treated with the Rigiflex balloon system (35 mm). In the remaining 5 (5/15) patients, a second dilation was not necessarry. One patient (1/16) was treated solely, but twice with the Rigiflex balloon system. The remaining three patients (3/19, 16%) were treated with Botox-injections.

In the subgroup of treated (SWS-negative) patients, median EGJ-DI was 2.1 mm^2^/mmHg (range 0.3–4.7 mm^2^/mmHg) and TBE (performed in 18/19 patients) showed stasis in 72%. Three of the treated patients had an EGJ-DI ≥ 3 mm^2^/mmHg. However, these patients showed stasis or abnormal motility during TBE, and reported an ES ≥ 4. Of the six patients that did not receive treatment, two patients (33%) showed an EGJ-DI ≥ 3 mm^2^/mmHg and an ES < 3. Three showed normal findings during TBE and/or ES < 3 and one patient showed a significant symptom reduction (ES = 0) following treatment with proton pump inhibitors and no stasis in TBE (EGJ-DI = 2.0 mm^2^/mmHg).

If performed (15/19, 79%), post-interventional TBE (performed in a median of 15 days post treatment; range 6–77) showed an improved outcome after completed treatment in all patients. In seven patients (47%) stasis in the 5 min image remained with reduced median height of 2 cm compared to 5 cm pre-interventionally. Eight patients (53%) showed no stasis after treatment, 5 of these had stasis and 3 abnormal motility and delayed passage pre-interventionally. In accordance, the post-interventional ES was significantly reduced with median values of 5 (range 1–10) and one (range 0–4) pre- and post-interventionally (*p* value < 0.001). Thirteen patients (69%) showed both a reduced ES of ≥ 3 points and an ES < 3 after completed therapy. Two patients (11%) showed an ES < 3 and another 2 (11%) a reduction of ≥ 3 points. Only 2 patients (11%) showed neither a reduction ≥ 3 points nor an ES of < 3 after completed therapy. In total, 17 of 19 patients (89%) responded to achalasia-type therapy.

## Discussion

This study was designed to further assess the diagnostic yield of both STM and EGJ-DI findings in non-achalasia, non-obstructive dysphagia patients with signs of esophagogastric junction outflow obstruction. We were able to demonstrate that STM and EGJ-DI can identify EGJOO in symptomatic patients with normal SWS during HRM. Adding STM to HRM led to achalasia-type treatment in 76% of SWS-negative patients with a clinical response rate of 89%.

This study is unique as it contains a group of patients with classical non-obstructive EGJOO according to CCv3 (SWS-group) and a group of symptomatic patients without signs of a major motility disorder during SWS (STM-group). In addition, all results were compared to asymptomatic patients (control group). This translates into the possibility of describing EGJ-DI values in EGJOO patients, which are diagnosed according to CCv3 [4], still regarded as state of the art in diagnostics of motility disorders, giving us the possibility to compare these values to those of symptomatic patients without the classical diagnosis of EGJOO (STM-group). By this, we were able to show that EGI-DI values < 3mm^2^/mmHg in case of uneventful standard HRM (SWS-negative) identifies EGJOO in symptomatic patients.

STM has been shown to increase the diagnostic sensitivity of HRM for the identification of major motility disorders in patients with dysphagia [7, 8], but has not been implemented into the diagnostic guidelines of dysphagia management. Ang et al. highlighted that the disorder most often not observed during SWS was EGJOO, even though 90% of these patients reported esophageal pressurizations during the investigation [7]. Wang et al. further supported the diagnostic yield of STM by demonstrating an increased rate of EGJOO identification in symptomatic patients after fundoplication [23].

STM has been implemented in the routine assessment of dysphagia in our clinic. Accordingly, STM-group resembles a group of patients, which, without the additional investigation of STM, would have been left without a diagnosis and thus unexplained symptoms and by this without a clear therapeutic recommendation. Due to the underlying study design, the rate of additional diagnoses of EGJOO by STM cannot be answered. However, 60% of patients of the analyzed cohort (25/42) were only diagnosed by elevated IRP during STM (= SWS-negative), leading to achalasia-type treatment in 19 patients (76%) as a consequence to the evaluation of STM. Due to a lack of specific documentation in our data base, a differentiation if a particular abnormal finding, rather than a combination of multiple results (STM ± FLIP ± TBE) led to the treatment decision was not possible. However, only due to the STM-findings in this patient group, further investigations were initiated, enabling interventional treatment in a very carefully selected patient cohort.

To the best of our knowledge, the underlying study is one of two studies [14] focusing on the evaluation of EGJ-DI in EGJOO patients with FLIP technology, which has previously been shown to be of value in the workup of achalasia and eosinophilic esophagitis [24–26]. Recently Triggs et al. demonstrated FLIP to be helpful in the assessment of EGJOO in terms of identification of patients most likely to benefit from achalasia-type treatment [14]. The EGJ-DI values of our patients identified with EGJOO during STM showed a median EGJ-DI of 2.1mm^2^/mmHg. FLIP investigation helped us to identify patients who benefited from achalasia-type treatment, as demonstrated by a significant reduction of symptoms (expressed as a reduction in ES) and esophageal stasis post-interventionally. These results are in line with findings of Triggs et al., who recently demonstrated that patients diagnosed with EGJOO according to CCv3 with EGJ-DI < 2 mm^2^/mmHg were three-fold more likely to have radiologic evidence (≥ 1 cm barium retention at 1 min) for an EGJ disorder on TBE [14]. In addition, the same group of patients showed positive results (in terms of a reduced ES) after achalasia-type therapy. In contrast, all patients with EGJ-DI > 3 mm^2^/mmHg (median, 7.3; range 4.9–7.9) improved with conservative treatment. The authors argue that those patients’ EGJ-DI were already within target range of achalasia-type treatment, making symptom relief as a consequence to a further reduction of EGJ outflow obstruction unlikely.

Not surprisingly, we found a significant difference in EGJ-DI between symptomatic and asymptomatic patients (Fig. 1). Further, we were able to demonstrate decreased EGJ-DI values in SWS-negative, but symptomatic patients (76% had an EGJ-DI < 3 mm^2^/mmHg). Median EGJ-DI values in the STM-group did not differ to those of EGJOO patients according to CCv3 (Fig. 2), demonstrating that the diagnosis of EGJOO during STM and SWS are comparable. The fact that STM-IRP showed a statistically significant association to EGJ-DI provides even further evidence that STM is able to identify EGJOO in case of uneventful SWS. In summary, our results further emphasize the diagnostic yield of FLIP and STM in assessing patients with dysphagia, since both investigations can identify EGJOO.

Today, no normative EGJ-DI values for the diagnosis of EGJOO exist. However, the clinical importance of EGJ-DI cannot be underestimated as its association with symptoms and esophageal retention (per TBE) may be stronger than conventional HRM findings [25]. In addition, in early stages of achalasia (and therefore EGJOO), endoscopy and TBE can be normal [27]. In accordance to the median EGJ-DI of 6.9 mm^2^/mmHg in asymptomatic patients found in this study, Carlson et al. reported a median EGJ-DI of 5.8mm^2^/mmHg in a prospective study of 20 asymptomatic volunteers [21]. Their conclusion of an EGJ-DI greater than 2.8mm^2^/mmHg to be normal, corresponding to results of earlier studies [13], goes along with our findings with a median EGJ-DI of 2.0 mm^2^/mmHg in all symptomatic patients.

In summary, in addition to the already established use in achalasia, FLIP technology seems to be of great diagnostic value in the management of SWS-negative dysphagia, with the potential of identifying a clinically relevant proportion of patients eligible for achalasia-type treatment.

This study is limited by its retrospective nature and the sample size, limiting its statistical power. In addition, the contractility pattern (absent, repetitive or retrograde) was not documented during all FLIP investigations and therefore no conclusions on the impact of EGJOO on esophageal contractility and resolution thereof can currently be made.

Our results emphasize the diagnostic yield of a standard test meal and FLIP in assessing patients with dysphagia. We not only described EGJ-DI values of classical EGJOO according to CCv3, but also of symptomatic patients with uneventful SWS evaluations during standard HRM. By this, we were able to demonstrate that FLIP may identify EGJOO in SWS negative patients, potentially during the index endoscopy. Last is not only of importance in terms of early diagnosis but also because many patients do not tolerate the HRM catheter. Moreover, the underlying study shows that STM increases the diagnostic sensitivity of HRM and helps diagnose EGJOO, resembling a cheap and valuable option whenever FLIP is unavailable or may help to carefully select symptomatic patients who should be referred to achalasia-type treatment.

With prospective, controlled studies pending, we believe that STM and FLIP are valuable tools in the assessment of non-achalasia and non-obstructive dysphagia patients.
